# Renal autotransplantation for pheochromocytoma involving the renal hilar vessels with kidney preservation: a case report and literature review

**DOI:** 10.3389/fonc.2026.1986562

**Published:** 2026-10-21

**Authors:** Xiaodong Chen, Xiaodong Fan, Guobin Weng, Jiangyong Lou

**Affiliations:** Department of Urology, Ningbo Integrated Traditional Chinese and Western Medicine Hospital, Ningbo, Zhejiang, China

**Keywords:** bench surgery, hypertensive crisis, kidney preservation, pheochromocytoma, renal autotransplantation

## Abstract

Pheochromocytomas are neuroendocrine tumors of the adrenal medulla that frequently secrete excessive catecholamines. When such tumors involve the renal hilar vessels, ipsilateral nephrectomy has traditionally been preferred, as *in situ* dissection risks major vascular injury and massive catecholamine release. This study presents a 37-year-old man with a left adrenal pheochromocytoma presenting with a hypertensive crisis (blood pressure 260/110 mmHg). Intraoperatively, the tumor was found to encase the left renal artery and adhere densely to the left renal vein, so the kidney could not be preserved *in situ*. The left kidney was removed for ex vivo cold perfusion and bench preparation, the tumor was resected *in situ*, and the kidney was autotransplanted into the left iliac fossa. Histopathology confirmed a pheochromocytoma with malignant potential. At the 2-year follow-up, blood pressure and renal function remained normal without antihypertensive medication, with no evidence of recurrence. Renal autotransplantation combined with ex vivo bench surgery is a feasible kidney-preserving strategy for pheochromocytomas involving the renal hilar vessels.

## Introduction

Pheochromocytomas are neuroendocrine neoplasms arising from the chromaffin cells of the adrenal medulla and frequently secreting excessive catecholamines, including epinephrine and norepinephrine. With an estimated prevalence of approximately 0.05%, these tumors occur predominantly in adults aged 30–50 yr (1). Patients classically present with the triad of episodic sweating, headache, and palpitations, most often accompanied by hypertension (2). If left undiagnosed or untreated, pheochromocytomas can precipitate catastrophic and potentially fatal complications, including hypertensive crisis, catecholamine crisis, cardiomyopathy, and multi-organ failure; metastatic disease develops in 10%–25% of cases, and 30%–40% of tumors harbor a germline predisposition (3, 4). Surgical resection remains the treatment of choice. However, for tumors located at the renal hilum that invade or encase the renal artery and vein, ipsilateral nephrectomy has traditionally been preferred to achieve hemostasis and complete excision (5, 6).

Renal autotransplantation combined with ex vivo bench surgery was initially developed for the management of complex renovascular disease and renal tumors, enabling lesions not amenable to *in situ* resection to be meticulously dissected under cold perfusion and subsequently reimplanted (7). However, published reports of this approach for pheochromocytoma involving the renal hilar vessels remain very rare. We report the case of a 37-yr-old man who presented as an emergency with a pheochromocytoma crisis. Intraoperative exploration revealed encasement of the left renal artery by the tumor and dense adhesion of the tumor to the left renal vein, so the tumor could not be separated *in situ*. The patient underwent ex vivo cold perfusion and bench preparation of the left kidney, *in situ* tumor resection, and autotransplantation into the left iliac fossa. At 2-yr follow-up, renal function remained normal, with no evidence of tumor recurrence. The diagnostic workup, perioperative management, and surgical strategy are discussed in the context of the published literature.

## Case presentation

A 37-year-old male patient was admitted to our emergency department on January 18, 2023, with a 16-h history of sudden-onset severe pain in the left upper quadrant. At the referring hospital, his blood pressure was recorded at 260/110 mmHg, and contrast-enhanced CT revealed a retroperitoneal mass adjacent to the left renal artery with resultant arterial narrowing; however, blood pressure control remained poor despite sodium nitroprusside infusion. Upon transfer to our center, pheochromocytoma-induced hypertension was diagnosed, and intravenous urapidil achieved partial blood pressure control.

Contrast-enhanced CT performed at our hospital demonstrated a 4.0 × 3.2 cm mixed-density mass in the left adrenal region with cystic degeneration, the solid components of which exhibited marked enhancement. The mass was indistinguishable from the left adrenal gland, and the adjacent left renal artery was compressed and attenuated, with obscuration of the intervening fat plane (**Figure 1A**).

**Figure 1:**
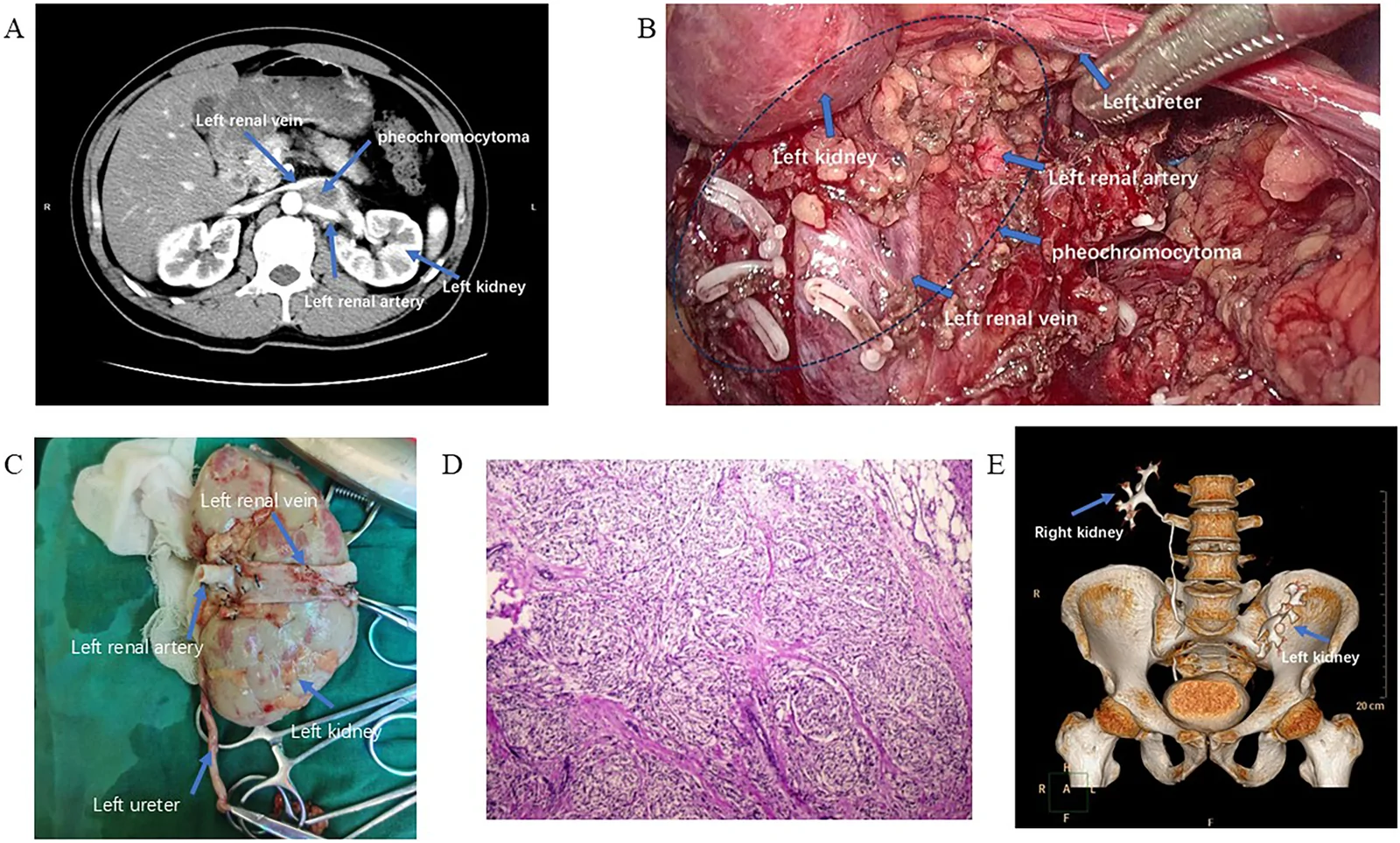
**(A)** Preoperative contrast-enhanced axial abdominal CT showing the left adrenal mass (pheochromocytoma) adherent to the left renal vein and encasing the left renal artery. **(B)** Intraoperative transperitoneal laparoscopic view showing the pheochromocytoma densely adherent to the left renal vein, with the left renal artery coursing through the tumor parenchyma; the vessels could not be dissected away from the tumor *in situ*. **(C)** Intraoperative bench photograph showing the explanted left kidney after cold perfusion, with the left renal artery, left renal vein, and left ureter trimmed and prepared for autotransplantation. **(D)** Postoperative histopathology showing tumor cells arranged in nests with comedo-like necrosis and cellular atypia. **(E)** Three-dimensional CT urography reconstruction at the 1-yr follow-up showing the right kidney *in situ* and the autotransplanted kidney in the left iliac fossa, both with intact contrast excretion, indicating preserved graft perfusion and function.

Laboratory examinations revealed markedly elevated supine renin (414.5 pg/mL; reference range: 4–24 pg/mL) and supine aldosterone (418.3 pg/mL; reference range: 10–160 pg/mL), whereas angiotensin II remained within normal limits, suggesting secondary activation of the renin–angiotensin–aldosterone system (RAAS) attributable to renal artery involvement. Serum creatinine was 67 μmol/L. The preoperative diagnosis was left adrenal pheochromocytoma with pheochromocytoma crisis.

Preoperative preparation consisted of oral terazosin 2 mg twice daily for 14 days, combined with metoprolol succinate 47.5 mg once daily; volume expansion was achieved with 1500 mL of intravenous normal saline daily alongside a regular diet. Blood pressure was maintained at 110–120/60–70 mmHg, and hemoglobin and hematocrit decreased from 162 g/L and 47.3% to 140 g/L and 39.9%, respectively, indicating effective volume restoration.

Surgery was performed on February 1, 2023. Transperitoneal laparoscopic exploration revealed dense adhesion of the tumor to the left renal vein, with the renal artery coursing through the tumor parenchyma, so the kidney could not be preserved *in situ* (**Figure 1B**); the procedure was therefore converted to an open transperitoneal approach through a left pararectus incision of approximately 20 cm. The left adrenal central vein was divided first, and the renal artery was ligated and divided proximal to its entry into the tumor. The left kidney, together with the ureter, was then removed after a warm ischemia time of 5 min and immediately flushed with 500 mL of 4 °C University of Wisconsin (UW) solution by gravity from a height of 1 m over 5 min, while a second surgical team trimmed the renal vessels and ureteral stump on the back table, where the kidney was kept in ice slush at 0 °C throughout bench preparation (**Figure 1C**). Meanwhile, the tumor was completely resected *in situ*, and intraoperative frozen-section examination confirmed pheochromocytoma. Following hemostasis and wound closure, the operative field was re-draped, and autotransplantation into the left iliac fossa was performed through an extraperitoneal approach via a curvilinear left lower abdominal incision: the renal artery was anastomosed end-to-end to the internal iliac artery, and the renal vein end-to-side to the iliac vein. Upon unclamping after a cold ischemia time of 90 min, the graft was immediately reperfused with visible urine output, and ureteroneocystostomy completed the procedure. The total operative time was 7 h, largely reflecting the prolonged attempt at *in situ* dissection before conversion, as no precedent for kidney preservation in this setting had been reported.

On postoperative day 1, serum creatinine was 91 μmol/L. Contrast-enhanced ultrasonography of the graft on postoperative day 2 demonstrated renal artery opacification at 7 s with good cortical perfusion and no segmental anastomotic stenosis (resistance index [RI] 0.81). Definitive histopathology revealed a 6.0 × 5.5 × 4.5 cm tumor composed of nests with comedo-like necrosis and cellular atypia, with focal extension into the periadrenal soft tissue; resection margins, capsular invasion, perineural invasion, and vascular invasion were all negative (**Figure 1D**). Immunohistochemistry showed positivity for chromogranin A (CgA), synaptophysin (Syn), and CD56, supporting neuroendocrine (chromaffin cell) origin, whereas steroidogenic factor-1 (SF-1) and pan-cytokeratin (pan-CK) were negative, excluding an adrenocortical origin; the Ki-67 index was 1% and phosphohistone H3 (PHH-3) was negative, indicating low proliferative activity. With a Pheochromocytoma of the Adrenal Gland Scaled Score (PASS) of 6 and a Grading System for Adrenal Pheochromocytoma and Paraganglioma (GAPP) score of 5, the tumor was diagnosed as a pheochromocytoma with malignant potential. The patient recovered uneventfully, with normalization of blood pressure and no further requirement for antihypertensive medication. At the 1-yr follow-up, serum creatinine was 70 μmol/L, and CT urography (CTU) showed good graft excretion without recurrence (**Figure 1E**). At the 2-yr follow-up, whole-abdomen CT revealed an unremarkable graft with no evidence of recurrence or metastasis, and renal function remained normal.

## Discussion

The present case featured an emergency presentation of pheochromocytoma crisis, with blood pressure reaching 260/110 mmHg accompanied by severe abdominal pain. Intraoperative exploration confirmed that the renal artery coursed through the tumor parenchyma and that the renal vein was densely adherent to the tumor, so the kidney could not be preserved *in situ*. The left kidney was therefore removed for ex vivo cold perfusion and bench preparation, while the tumor was completely resected *in situ*, followed by autotransplantation into the left iliac fossa. At the 2-yr follow-up, renal function remained normal, with no evidence of recurrence or metastasis, supporting the mid-term efficacy of this strategy.

When pheochromocytomas and renal hilar paragangliomas involve the renal vasculature, surgical management has long been trapped in a dilemma between radical resection and kidney preservation: dissection of tumors closely apposed to the renal vessels carries a constant risk of tearing the renal artery or vein, or of provoking massive catecholamine release into the circulation. Consequently, most reported cases have been managed with ipsilateral nephrectomy (5, 6).

Renal autotransplantation could solve this dilemma, but the published evidence is very limited. Gill et al. reviewed 269 cases of pheochromocytoma at their center, of which only 10 had coexisting renal artery lesions and only 1 underwent renal autotransplantation—and even that single case was performed for renal artery stenosis rather than tumor invasion (8). To our knowledge, renal autotransplantation with kidney preservation for pheochromocytoma involving the renal hilar vessels has not been reported to date; in complex renal tumor surgery, Xu et al. reported six cases of nephrectomy, bench surgery, and autotransplantation with no postoperative dialysis and no significant increase in serum creatinine at 30 days (7). In our patient, the renal artery coursed through the tumor parenchyma and the renal vein was densely adherent to the tumor, so *in situ* preservation of the kidney was impossible. Renal autotransplantation thus became the only option reconciling radical resection with kidney preservation. At the 2-yr follow-up, renal function remained normal and no tumor recurrence was observed, supporting the value of this approach.

Several technical points are worth noting. Early division of the adrenal central vein and the renal artery minimizes catecholamine release into the circulation provoked by tumor manipulation. Another point is the two-team workflow: after kidney retrieval, the primary surgical team continued with the *in situ* tumor resection, while a second team simultaneously trimmed the renal vessels and ureteral stump under cold perfusion on the back table, so tumor excision proceeded without interference from the kidney-preserving maneuvers, and the kidney remained under cold protection throughout. Finally, the autotransplantation itself used a conventional iliac fossa technique—end-to-end anastomosis of the renal artery to the internal iliac artery and end-to-side anastomosis of the renal vein to the iliac vein—both well-established procedures that require no specialized instruments and can be performed in centers with vascular surgery expertise.

Histopathologically, our patient had a PASS score of 6 and a GAPP score of 5 (moderately differentiated), with focal invasion of the periadrenal soft tissue. According to the 2022 World Health Organization (WHO) classification, pheochromocytomas and paragangliomas are no longer categorized as benign or malignant, and a diagnosis of metastatic disease can only be established when metastases occur; however, tumors harboring such histological features carry an increased risk of recurrence and metastasis, and postoperative recurrence may be delayed by many years (9, 10). Although no recurrence was observed during the 2-yr follow-up in our patient, continued long-term surveillance remains warranted.

This case has its limitations: preoperative biochemical testing for catecholamines was not completed, and the diagnosis was established on the basis of the typical crisis presentation, imaging findings, and histopathology. In conclusion, for pheochromocytomas involving the renal hilar vessels, ex vivo cold perfusion and bench preparation of the kidney, combined with *in situ* tumor resection and subsequent renal autotransplantation, can preserve renal function while achieving radical tumor resection, with satisfactory mid-term outcomes. This strategy is technically demanding in terms of vascular surgery and transplantation expertise, and patients who are young, harbor a solitary kidney, or have impaired contralateral renal function stand to benefit the most; its long-term efficacy awaits validation in additional cases.

## Data Availability

The original contributions presented in the study are included in the article/supplementary material. Further inquiries can be directed to the corresponding authors.

## References

[1] Strosberg JR . Update on the management of unusual neuroendocrine tumors: Pheochromocytoma and paraganglioma, medullary thyroid cancer and adrenocortical carcinoma. Semin Oncol. (2013) 40:120–33. doi: 10.1053/j.seminoncol.2012.11.009 23391119

[2] Saavedra TJ Nati-Castillo HA Valderrama Cometa LA Rivera-Martinez WA Asprilla J Castano-Giraldo CM . Pheochromocytoma: An updated scoping review from clinical presentation to management and treatment. Front Endocrinol (Lausanne). (2024) 15:1433582. doi: 10.3389/fendo.2024.1433582 39735644 PMC11671257

[3] Lenders JW Duh QY Eisenhofer G Gimenez-Roqueplo AP Grebe SK Murad MH . Pheochromocytoma and paraganglioma: An endocrine society clinical practice guideline. J Clin Endocrinol Metab. (2014) 99:1915–42. doi: 10.1210/jc.2014-1498 24893135

[4] Sharma S Fishbein L . Diagnosis and management of pheochromocytomas and paragangliomas: A guide for the clinician. Endocr Pract. (2023) 29:999–1006. doi: 10.1016/j.eprac.2023.07.027 37586639

[5] Kamali K Rajabi M Padooiy Nooshabadi M . Renal hilar paraganglioma with atypical clinical presentation and biochemical functionality: A case report of diagnostic pitfalls. Urol Case Rep. (2026) 68:103567. doi: 10.1016/j.eucr.2026.103567 42620445 PMC13485497

[6] Sathaye S Parmar KM Kumar S Rastogi P . Adrenal pheochromocytoma with involvement of aorta and left renal artery: Salvaged with adrenalectomy and nephrectomy. BMJ Case Rep. (2021) 14:e244297. doi: 10.1136/bcr-2021-244297 34511410 PMC8438742

[7] Xu Y Huang J Fan X Wang Z Lou J Liu X . Clinical experience of bench surgery combined with autotransplantation after three-dimensional laparoscopic nephrectomy for the treatment of highly complex renal tumor. World J Surg Oncol. (2023) 21:373. doi: 10.1186/s12957-023-03246-9 38031058 PMC10687882

[8] Gill IS Meraney AM Bravo EL Novick AC . Pheochromocytoma coexisting with renal artery lesions. J Urol. (2000) 164:296–301. doi: 10.1016/s0022-5347(05)67344-2 10893569

[9] Mete O Asa SL Gill AJ Kimura N de Krijger RR Tischler A . Overview of the 2022 Who classification of paragangliomas and pheochromocytomas. Endocr Pathol. (2022) 33:90–114. doi: 10.1007/s12022-022-09704-6 35285002

[10] Holscher I van den Berg TJ Dreijerink KMA Engelsman AF Nieveen van Dijkum EJM . Recurrence rate of sporadic pheochromocytomas after curative adrenalectomy: A systematic review and meta-analysis. J Clin Endocrinol Metab. (2021) 106:588–97. doi: 10.1210/clinem/dgaa794 33125073

